# Effect of an Olive Vegetation Water Phenolic Extract on the Physico-Chemical, Microbiological and Sensory Traits of Shrimp (*Parapenaeus longirostris*) during the Shelf-Life

**DOI:** 10.3390/foods9111647

**Published:** 2020-11-11

**Authors:** Dino Miraglia, Marta Castrica, Laura Menchetti, Sonia Esposto, Raffaella Branciari, David Ranucci, Stefania Urbani, Beatrice Sordini, Gianluca Veneziani, Maurizio Servili

**Affiliations:** 1 Department of Veterinary Medicine, University of Perugia, Via San Costanzo 4, 06126 Perugia, Italy; dino.miraglia@unipg.it (D.M.); raffaella.branciari@unipg.it (R.B.); david.ranucci@unipg.it (D.R.); 2 Department of Health, Animal Science and Food Safety “Carlo Cantoni”, Università degli Studi di Milano, Via Celoria 10, 20133 Milan, Italy; marta.castrica@unimi.it; 3 Department of Agricultural and Agri-Food Sciences and Technologies, University of Bologna, Viale Fanin 46, 40138 Bologna, Italy; laura.menchetti@unibo.it; 4 Department of Agricultural, Food and Environmental Sciences, University of Perugia, Via San Costanzo s.n.c., 06126 Perugia, Italy; stefania.urbani@unipg.it (S.U.); beatrice.sordini@unipg.it (B.S.); gianluca.veneziani@unipg.it (G.V.); maurizio.servili@unipg.it (M.S.)

**Keywords:** olive by-product, olive phenolic extract, anti-lipid oxidation effect, antibacterial activity, melanosis, additive-free shrimp preservation

## Abstract

The aim of this study was to evaluate the effects of phenolic extract derived from olive vegetation water (PEOVW) in deep-water rose shrimps (*Parapenaeus longirostris*) at the day of packaging (D0) and after three (D3), six (D6) and eight (D8) days of refrigerated storage. Freshly caught shrimps were randomly divided into four groups: the phenolic extract (PE) group (2 g/L of phenols); the sulfites (S) group (0.5% sodium metabisulfite solution); the phenolic extract + sulfites (PE + S) group (0.25% sodium metabisulfite solution with 1 g/L of phenols), and the control (CTRL) group (tap water). Concerning color coordinates, there were no variations either between groups or over time, while it is important to highlight that phenolic extract (PE group) led to a significant reduction in total volatile basic nitrogen (TVB-N; *p* < 0.001) and thiobarbituric reactive substances (TBARS; *p* < 0.001) values. Furthermore, PE also had a relevant effect in reducing bacterial counts and decreasing the microbial development. Finally, as concerns melanosis, the effect of phenolic extract alone was marginal, but when combined with half a dose of sodium metabisulfite, it was as effective as the shrimps treated with only sodium metabisulfite in delaying black spots (*p* < 0.05). These results are very promising with a view to commercializing additive-free shrimps.

## 1. Introduction

The deep-water rose shrimp (*Parapenaeus longirostris*) is one of the most important commercial species of the Mediterranean coast for many countries, including Spain, France and Italy. However, it has a wide geographic distribution and the interest in this species is also growing in many developing countries [1,2,3]. Shrimps are appreciated for their high nutritional value, since they are rich in poly unsaturated fatty acids, and have unique sensory characteristics [3].

Unfortunately, melanosis and microbiological deterioration are common problems during the post-harvest storage of crustaceans, damaging their sensory features, and decreasing their quality, shelf-life, and subsequently, their commercial value [4]. In order to delay deteriorative processes and reduce the economic losses, different methods have been studied. Nowadays, the additives most widely used are the sulfites, especially sodium metabisulfite. The immersion in a blend of sulfite derivate immediately after capture is effective in preventing the melanosis from reacting with intermediate quinones in the melanosis reaction, and inactivating the polyphenoloxidase (PPO), a key enzyme in the synthesis of melanin [4,5]. Sulfites also have a significant antimicrobial activity, for example against *Aspergillus* spp. and *Penicillium* spp. [4].

Nevertheless, sulfites could accumulate above the maximum residue limit prescribed by the law and have potentially pathological effects, such as asthma and skin allergy [6,7]. There is, then, increasing interest in natural alternatives to the antimelanosic chemical agents, responding to consumers’ requirements and food safety regulations [4,8]. The phenolic compounds could be important candidates thanks to their antioxidant and antimicrobial activities, and can act in different ways [9]. They can interact with microbial membrane proteins, enzymes and lipids, thereby altering cell permeability, they can cross the membrane and act against enzymes and proteins, and they could interfere with the membrane function, deforming its structure [10]. Moreover, they are substances of natural origin, that the consumer tends to accept more willingly than the synthetic chemical ones [11,12].

Phenolic compounds, including tocopherols, flavonoid compounds, cinnamic acid derivatives and coumarins, can be easily obtained from several natural sources, such as fruits, vegetables, coffee, tea, beer, wine and oil [10,13]. During the past decade, the use of plant-derived phenolic compounds has been evaluated in different fish products, including shrimp. Nirmal and Benjakul [14,15] found that different phenolic compounds, as well as green tea extract, improved the quality of the shrimp during storage. Other authors have obtained the inhibition of melanosis in shrimp using grape seed extracts [8,16]. Olives and their derivates are rich in phenolic compounds (Table S1), their antioxidant and nutraceutical properties are well known [9], and their production is spread in the Mediterranean area. Precisely because of their antioxidant power combined with antibacterial activity [17,18,19], they are used to improve the conservation and quality of meat and fruit [11,20,21], but, to our knowledge, there is only one report investigating their effects on the shelf-life of shrimp [22]. These authors suggested that immersion in a solution with olive leaf extract for 3h improves the microbial quality of Green tiger shrimp (*Penaeus semisulcatus*). The antibacterial activity was concentration-dependent, and the study only provided bacteriological evaluations. However, it showed the potential use of olive derivatives during shrimp processing [22]. Among olive by-products, the use of vegetation waters in food application is one of the most demanding and urgent challenges. Indeed, they contain many phenols which have several health-beneficial properties [13,23,24]. However, to date, they represent only an economic burden to producers due to the negative environmental effects induced by their disposal [21,25]. In this regard, according to Souilem et al. [26], the annual yield of olive vegetation water (OVW) worldwide is estimated to be from 10 to over 30 million m^3^.

The aim of this study was to evaluate the effects of a phenolic extract derived from olive vegetation water (PEOVW) on the shelf-life of deep-water rose shrimp. Chemical, microbial and sensorial changes were evaluated in shrimp during storage at 2 °C, pre-treated with phenolic extract and a sulfites derivate.

## 2. Materials and Methods

### 2.1. Phenolic Extract and Shrimps Sample

The phenolic extract (Figure S1) used for the experimentation was obtained from olive vegetation water (OVW; Table S1) as described by Esposto et al. [27] and stored at −20 °C until its use. The extract, at the moment of its utilization, contained 520.9 mg/g total phenolics represented by 106.6 mg/g of 3,4-dihydroxyphenylethanol (3,4-DHPEA), 19.1 mg/g of *p*-hydroxyphenylethanol (*p*-HPEA), 31.5 mg/g of verbascoside and 363.7 mg/g of the dialdehydic form of elenolic acid linked with 3,4-dihydroxyphenylethanol (3,4-DHPEA-EDA).

Freshly caught deep-water rose shrimps (*Parapenaeus longirostris*) were purchased, without any treatment, from the supplier based in Civitanova Marche, in the province of Macerata, Italy. Shrimps were kept on ice and transported to a fish processing factory in Perugia, Italy. Upon arrival, shrimps were randomly divided into 4 groups and immersed for 15 min at 12 °C in the following solutions (2/1 solutions/shrimp):

- tap water (control group, CTRL);
- tap water solution containing 2 g/L of phenols (phenolic extract group, PE) obtained by adding 1.92 g PEOVW/L;
- 0.5% sodium metabisulfite tap water solution (sulfites group, S);
- 0.25% sodium metabisulfite tap water solution containing 1 g/L of phenols (phenolic extract + sulfites group, PE + S) obtained by adding 3.84 g PEOVW/L.

The shrimps were then drained and arranged into 4 polystyrene containers (300 g/container) for each group and packed in an oxygen-permeable package. While being stored under refrigeration, the shrimps were transported to the Department of Veterinary Medicine and stored at 2 °C. Samples were randomly analyzed for microbiological, chemical, physical, and sensory characteristics at the following times: the day of packaging (D0), and three (D3), six (D6) and eight (D8) days after packaging. 

### 2.2. Phenolic Extraction and HPLC Analysis

Phenolic extraction and evaluation were carried out according to the method described by Miraglia et al. [18] with slight modifications as explained below. Five grams of muscle was homogenized with 50 mL of methanol + water (80:20% *v*/*v*) + butylated hydroxytoluene (20 mg/L)+ trichloroacetic acid (2 M, with a final proportion in the solution of 0.2%) using an Ultraturrax^®^T50 apparatus (Janke and Kunkel, Staufen, Germany) at 7000 rpm for 1 min. The homogenized product was centrifugated at 9000 rpm for 10 min. The extraction was repeated twice. The supernatant was transferred and collected in a flask. The solvent was removing at 37 °C using a rotavapor, until the final volume of the aqueous extract was 20 mL. Solid phase extraction (SPE) was applied to extract the phenolic compounds. Then 15 mL samples of the aqueous extract were loaded into SPE HF Mega Bond Elut-C18, 5 g cartridges (Agilent Technologies, Santa Clara, CA, USA), after its activation with methanol and water, and 100 mL of methanol was applied to elute the phenolic compounds. The elute was dried at 37 °C using a rotavapor, and the dried product was solubilized using 4 mL of methanol which was then evaporated under N2 flushing. For the HPLC analysis, the dried extract was recovered with 0.5 mL of methanol and filtered with 0.2 μm polyvinylidene fluoride (PVDF) filters (Whatman, Clifton, NJ, USA), and then 20 μL were injected into an HPLC instrument equipped and set up as reported for the analytical determination of the hydrophilic phenols of phenolic extract [28].

### 2.3. Instrumental Analysis: Color and pH

Color, expressed as lightness (L*), yellowness (b*) and redness (a*), of cephalothorax carapace was evaluated with a colorimeter (Minolta Chromameter 400; Minolta Ltd., Osaka, Japan), using a CIE L* a* b* scale. The pH values were recorded using a pH meter (Crison pH25, Crison, Barcelona, Spain). All analyses were performed in triplicate.

### 2.4. Chemical Analyses

The total volatile basic nitrogen (TVB-N) was determined using VELP Marka model UDK 139 apparatus (Velp Scientifica, Usmate, Milan, Italy). To briefly illustrate, homogenized shrimp samples were alkalized with 2 g of magnesium oxide and, subsequently, the TVB-N value (mg of N per 100 g of shrimp meat) was determined by steam distillation and titration with 0.01 N HCl [29]. The thiobarbituric reactive substances (TBARS), expressed as mg malondialdehyde (MDA)/kg muscle, were measured to evaluate the lipid oxidation [30].

### 2.5. Microbiological Analyses

Five samples of 10 g each were collected aseptically and were placed in a stomacher bag containing 90 mL of saline water. After mixing for 1 min in a Stomacher blender (Stomacher 400, Seward Ltd., Norfolk, UK), further serial dilution was done using the same diluent. Total viable count (TVC) and psychrotrophic bacterial count were determined using Plate Count Agar (PCA, Oxoid, Basingstoke, UK) and the plates were incubated at 30 °C for 48 h and 4 °C for 10 days, respectively [18,31]. Violet Red Bile Glucose agar (VRBG, Oxoid), incubated at 37 °C for 24 h, was used for *Enterobacteriaceae* count [18], while for *Pseudomonas* spp. count we used Pseudomonas agar base (Oxoid) with added CFC (Biolife Italiana s.r.l., Milano, Italy), and the plates were incubated at 25 °C for 48 h as described by Sae-leaw and Benjakul [31]. All microbiological counts were expressed as the log of the colony forming units per gram (Log CFU/g) of the sample. All analyses were performed in duplicate.

### 2.6. Melanosis Assessment

Twelve trained PANELLISTS evaluated melanosis in raw shrimps according to the Sae-leaw and Benjakul [31] method. Melanosis, manifested as black spots on the shell, was assessed using a visual scale from 1 to 10, where 0 = complete absence of melanosis, 2 = slight melanosis (up to 20% of the shrimps’ surface affected), 4 = moderate melanosis (20 to 40% of the shrimps’ surface affected), 6 = evident melanosis (40 to 60% of the shrimps’ surface affected), 8 = severe melanosis (60 to 80% of the shrimps’ surface affected), and 10 = very severe melanosis (80 to 100% of the shrimps’ surface affected).

### 2.7. Statistical Analyses

Microbiological and physico-chemical parameters were analyzed by using the linear mixed models (LMM), in which the time (day after packaging) was included as the repeated factor with a first-order autoregressive covariance structure. Time (4 levels: D0–D3) and group (4 levels) were evaluated in full factorial models. Diagnostic graphics were used to check assumptions. Logarithmic transformation was used for L*, a* and b* values. Data were presented as estimated marginal means, and Sidak adjustment was used for carrying out multiple comparisons. A nonparametric approach was chosen for melanosis and the scores of each evaluator were treated as paired data. At each time, the effect of the group was analyzed using the Friedman test followed by Dunn’s multiple comparisons test.

Moreover, for each group, the kinetic parameters of bacterial growths were calculated assuming a log-linear response based on the momentary survival ratio:

S(*t*) = N(*t*)/N0 (1)

where N(*t*) and N0 are the momentary and the initial number of microorganisms.

Then, the model resulted in:

Ln S(*t*) = k *t* (2)

where the growth rate constant (k) was calculated from the slope of the line via regression analysis of ln S(*t*) versus time and the “generation time” (τ, i.e., the time needed to double the population) as ln 2/k [32,33]. 

Finally, correlations were evaluated by using the Spearman rank correlation coefficient (*ρ*). The correlation was defined as high when the absolute value of was *ρ* > 0.5, medium when *ρ* ranged from 0.3 to 0.5, and low when *ρ* < 0.3 [34]. Statistical analyses were performed with SPSS Statistics version 25 (IBM, SPSS Inc., Chicago, IL, USA). Statistical significance occurred when *p* < 0.05.

## 3. Results and Discussion

### 3.1. Phenolic Compound Assessment

**Figure 1:**
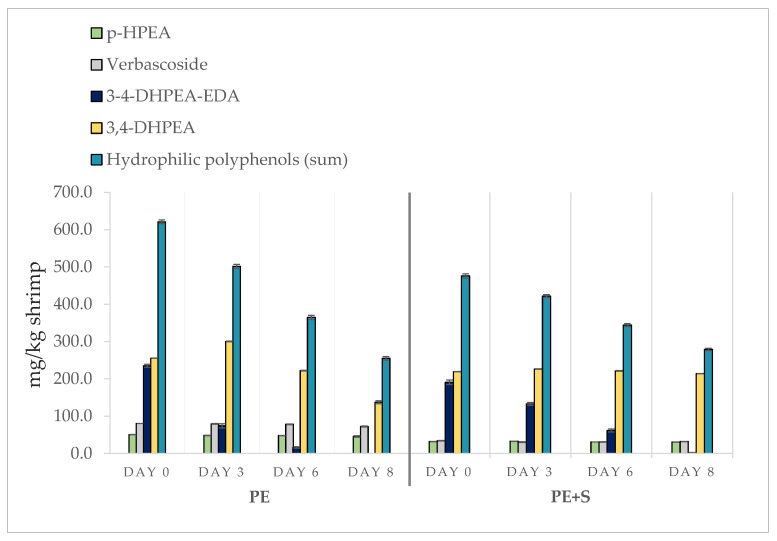
Trend of phenolic compounds during storage in shrimps (*n* = 5) treated with tap water solution containing 2 g/L of phenols (PE), and 0.25% sodium metabisulfite tap water solution containing 1 g/L of phenols (PE + S). Results are expressed as the sum of 3,4-DHPEA, *p*-HPEA, 3,4-DHPEA-EDA and verbascoside (mg/kg), and as individual phenolic compounds.

The phenolic compounds detected in the samples after treatment with PEOVW at different concentrations, and their evolution over time, are shown in Figure 1. The amounts of polyphenols recovered in shrimp tissues were proportional to the concentrations of the individual compounds in the solutions, but the absorption percentages varied according to the phenolic fraction considered and the starting solution. The most absorbed molecules were those with the lowest molecular weight, which were 3.4-DHPEA (107.2% in PE + S group and 62.4% in PE group) and *p*-HPEA (86.4% in PE + S group and 68.7% in PE group), while 3,4-DHPEA-EDA, characterized by a high molecular weight, was absorbed in lower percentages (27.4% and 16.8% in PE + S and PE groups, respectively). The higher dimension of this molecule probably makes the penetration into the tissues slower [18]. Interestingly, apart from verbascoside (56.1% and 66.3% in PE + S and PE groups, respectively), the highest absorption rates were always found in PE + S samples. This can be explained by the antioxidant action exercised by sodium metabisulfite on polyphenols, which reduced the oxidation of the phenols themselves. The protective effect of sulfites is also highlighted by the trend of phenolic compounds over time with minor decreases in PE + S shrimps (Figure 1). At the end of the storage period, the total phenolic content recovered was 41.1% and 58.6% of the initial concentration in PE and PE + S groups, respectively. In particular, 3,4-DHPEA-EDA was the fraction that decreased the most, disappearing totally on the eighth day in the PE group. This rapid decrease, that had already started on the third day, is to be attributed both to the involvement of the molecule in contrasting oxidative processes and to the hydrolysis phenomena, responsible, moreover, for the initial increase recorded for the 3,4-DHPEA [27,35], allowing the latter to achieve a higher concentration than those present in the PE + S solution (107.2%). No traces of the phenolic compounds were found in the CTRL and S groups.

### 3.2. Physical and Chemical Quality Analysis

**Figure 2:**
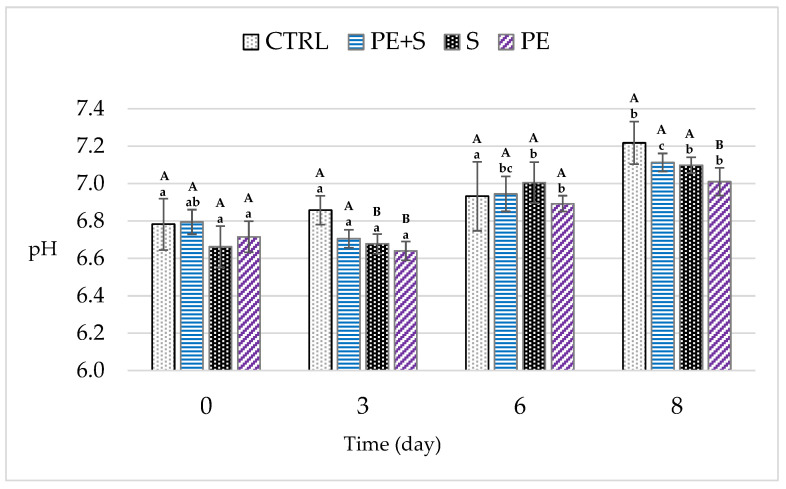
Changes in pH values during storage (D0 = day of packaging, D3 = three days after packaging, D6 = five days after packaging, and D8 = eight days after packaging) of shrimps (*n* = 5) treated with tap water (CTRL), 0.25% sodium metabisulfite tap water solution containing 1 g/L of phenols (PE + S), 0.5% sodium metabisulfite tap water solution (S) and tap water solution containing 2 g/L of phenols (PE). Values are means ± standard error of the means. Different uppercase letters, within each day of storage, represent significant differences between groups (*p* < 0.05); different lowercase letters, within each group, represent significant differences between days of storage (*p* < 0.05).

Overall, even though the shrimps’ pH always remained below the limits of acceptability (7.6) [36,37], it progressively increased during storage by 0.37 ± 0.03 units (Figure 2; *p* < 0.001), reaching values ranging from 7.01 ± 0.04 to 7.22 ± 0.04 eight days after packaging for the PE and CTRL groups, respectively. The latter showed higher values than those for the treated shrimps at D3 (*p* < 0.05), but at the end of the storage period only the PE group had a lower pH than the control (*p* < 0.01). In all likelihood, the phenolic compounds, controlling the microbial growth, lead to a reduction in the TVB-N amount in the PE group [18]. As is known, in fact, the pH rise is due to the accumulation of basic compounds, such as amines and trimethylamines, because of endogenous and microbial enzymatic action [38]. Furthermore, in our study, total viable count, psychrotrophic bacteria and TVB-N were correlated with the pH at day 8 (*p* < 0.01; Table S2).

Trimethylamine, dimethylamine, ammonia and other volatile nitrogenous compounds were evaluated by the TVB-N [39]. The European Regulation No. 2074/2005 establishes limit values of TVB-N for certain fish species; indeed, TVB-N is the spoilage index most commonly used to assess the quality of fish [40].

**Figure 3:**
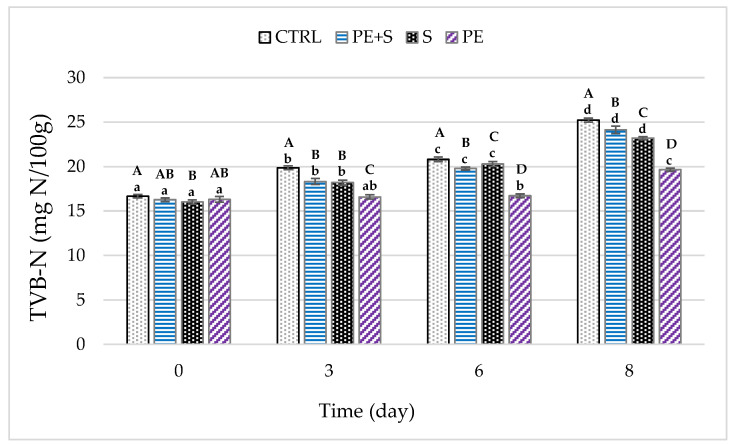
Changes in TVB-N (total volatile basic nitrogen) values during storage (D0 = day of packaging, D3 = three days after packaging, D6 = five days after packaging, and D8 = eight days after packaging) of shrimps (*n* = 5) treated with with tap water (CTRL), 0.25% sodium metabisulfite tap water solution containing 1 g/L of phenols (PE + S), 0.5% sodium metabisulfite tap water solution (S) and tap water solution containing 2 g/L of phenols (PE). Values are means ± standard error of the means. Different uppercase letters, within each day of storage, represent significant differences between groups (*p* < 0.05); different lowercase letters, within each group, represent significant differences between days of storage (*p* < 0.05).

Regardless of the treatment, TVB-N mean values increased from 16.3 ± 0.1 mg N/100 g on the day of packaging (D0) to 23.0 ± 0.1 mg N/100 g after eight days (*p* < 0.001). In particular, the values at D0 ranged from 16.02 to 16.66 mg N/100 g for the S and CTRL groups, respectively (S vs. CTRL: *p* < 0.01), while the PE and PE + S groups did not differ from the CTRL group (*p* > 0.05) (Figure 3). As conservation continued, the differences between CTRL shrimps and treated groups increased. In fact, the multiple comparisons indicated that beginning with the second detection (D3), the CTRL group always maintained higher mean values compared to the other groups (*p* < 0.01). Therefore, all the treatments tested were able to delay the formation of TVB-N, although with a differentiated efficacy. Indeed, the trend in TVB-N over time showed a significant increase at each step of the analysis, with the exception of the PE group, where the amount of TVB-N remained stable until the sixth day of storage (*p* > 0.05). In percentage terms during storage, the TVB-N values grew by 51% (8.55 ± 0.1 mg N/100) in CTRL and only by 20% (3.33 ± 0.1 mg N/100) in the PE group. Consequently, at the end of the observation period, the lowest mean values were found in the PE group, followed by the S and PE + S groups (*p* < 0.001). 

As is known, the formation of volatile nitrogenous compounds is a result of microbial and enzymatic activities, and their post-mortem accumulation involves sensory and chemical–physical alterations with a consequent quality loss of the products [29]. To confirm this, in the current work the TVB-N levels at day 8 were strongly positively correlated with the total viable count, psychrotrophic counts and pH (*p* < 0.01; Table S2). Other authors reported similar or higher TVB-N values, suggesting that our shrimps were of good quality [36,38,41].

The above results agree with those of previous studies which reported a lower increase in TVB-N values in the shrimps treated with a chitosan coating in comparison with the control through storage [42,43,44]. Furthermore, Yuan et al. [42] highlighted a synergistic effect in the delayed TVB-N value when the chitosan coating was used in combination with green tea extract. Similarly, other authors reported that the TVB-N values of Pacific white shrimp treated with ferulic acid, cinnamaldehyde and grape seed extract were significantly decreased [15,16,45].

**Figure 4:**
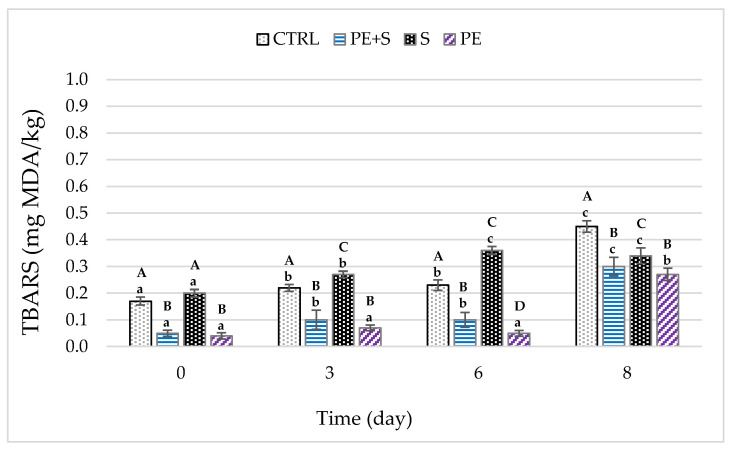
Changes in TBARS (thiobarbituric reactive substances) values during storage (D0 = day of packaging, D3 = three days after packaging, D6 = five days after packaging, and D8 = eight days after packaging) of shrimps (*n* = 5) treated with tap water (CTRL), 0.25% sodium metabisulfite tap water solution containing 1 g/L of phenols (PE + S), 0.5% sodium metabisulfite tap water solution (S), and tap water solution containing 2 g/L of phenols (PE). Values are means ± standard error of the means. Different uppercase letters, within each day of storage, represent significant differences between groups (*p* < 0.05); different lowercase letters, within each group, represent significant differences between days of storage (*p* < 0.05).

The tissues of crustaceans are, of course, particularly susceptible to lipid oxidation because they possess high amounts of polyunsaturated fatty acids. Lipid oxidation creates severe quality problems both from a nutritional and commercial point of view [15,46,47], and can be due to both enzymes such as lipoxygenase and peroxidase, and microsomal enzymes [48]. Lipid oxidation can be assessed through TBARS. Overall, the TBARS values increased as storage time increased (*p* < 0.001), but they remained below the threshold proposed by Schormüller and Bonnell [49,50] (<5 and <2 mg malonaldehyde/kg, respectively) for good quality fish. Moreover, the two groups treated with the phenolic extract (PE and PE + S) showed lower values compared to all the other samples already at D0 (Figure 4). This result suggests that phenolic extract limited the lipid oxidation in shrimp muscle starting from the time of packaging. After eight days, the TBARS values of the shrimps treated with phenolic extract were 40% and 33% lower than the control group for PE and PE + S, respectively (*p* < 0.001).

The lowered lipid oxidation of shrimps treated with sulfites and/or phenolic extract can be attributed to their ability to scavenge free radicals and/or to chelate prooxidative metal ions [10]. In addition, these findings are in accordance with the lowered microbial growth of treated groups. In the present study, the TBARS values correlated positively with TVC (*p* < 0.05), *Enterobacteriaceae* (*p* < 0.01) and psichrotrophic bacterial count (*p* < 0.01) (Table S2). In particular, the latter produces lipase and phospholipase, causing an increase in free fatty acid highly susceptible to oxidation [31,51]. A drop in the pH, TVB-N and TBARS of shrimps was previously obtained by using green tea and modified atmosphere packaging [15], or ferulic acid [14]. Grape seed extracts [16] and cinnamaldehyde [45] changed the pH and TVB-N of shrimp, but TBARS values were not evaluated.

Cadun et al. [52] found that the addition of rosemary extract to a marination treatment with NaCl and organic acids reduced TVB-N and TBARS values, but it did not change the shrimp’s pH values during storage. Conversely, López-Caballero et al. [41] found a slight increase in TVB-N using a formulation containing 4-hexylresorcinol, chelating agents and acids.

**Figure 5:**
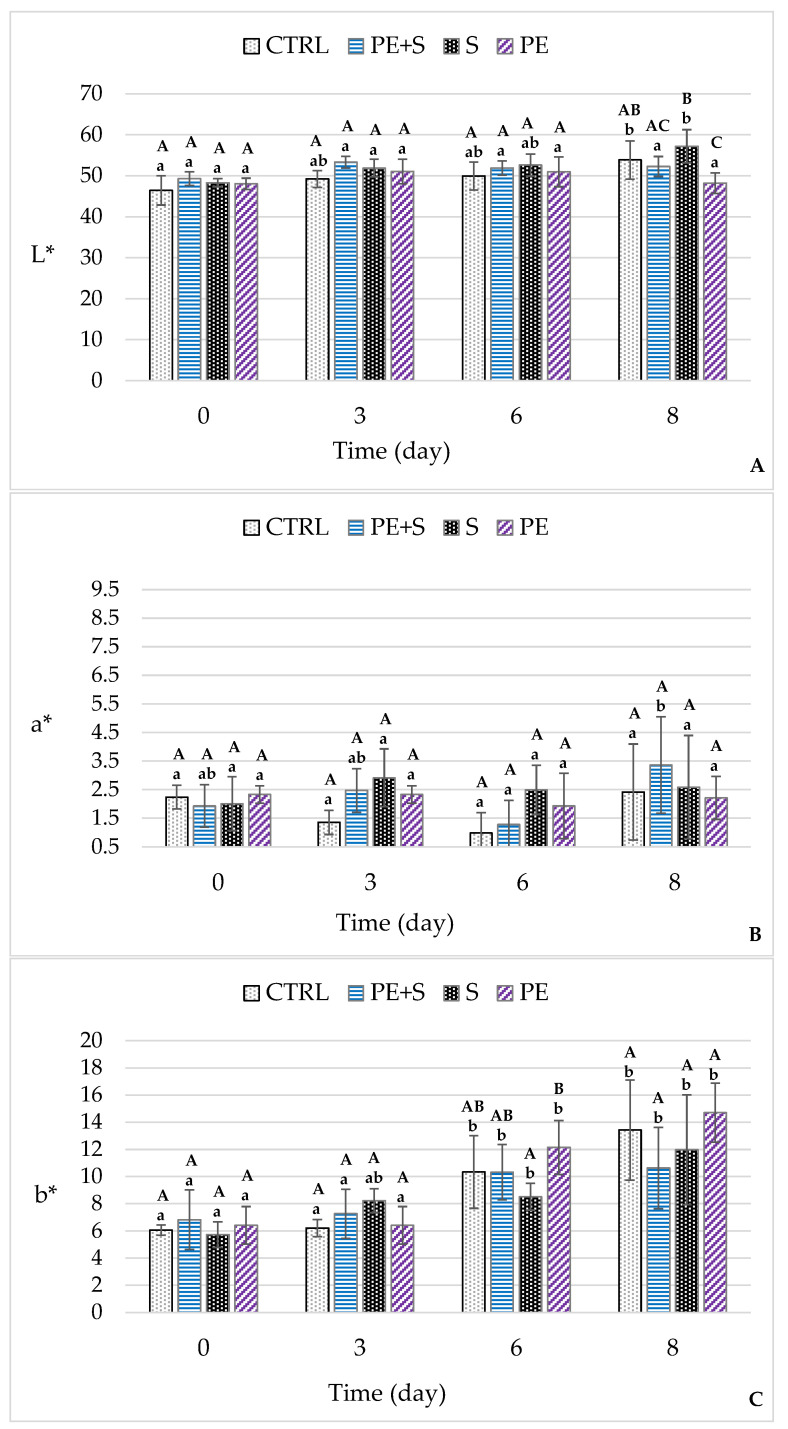
Changes in color values L* (**A**), a* (**B**) and b* (**C**), during storage (D0 = day of packaging, D3 = three days after packaging, D6 = five days after packaging, and D8 = eight days after packaging) of shrimps (*n* = 5) treated with tap water (CTRL), 0.25% sodium metabisulfite tap water solution containing 1 g/L of phenols (PE + S), 0.5% sodium metabisulfite tap water solution (S) and tap water solution containing 2 g/L of phenols (PE). Values are means ± standard error of the means. Different uppercase letters, within each day of storage, represent significant differences between groups (*p* < 0.05); different lowercase letters, within each group, represent significant differences between days of storage (*p* < 0.05).

Overall, color parameters are associated with melanosis [8,38]. Indeed, in general, while melanosis increases over time, lightness and redness tend to decrease, and yellowness increases in parallel [41]. Nevertheless, in our study, the L* coordinate assessment did not highlight any changes over time except for an increase in the CTRL and S groups at the end of the observation period compared to at three and six days, respectively (Figure 5A). Furthermore, the treatment effect seems to have occurred only on day eight, when the shrimps belonging to the S group were brighter than those of the PE and PE + S groups, but not those of the control group. 

As regards the a* coordinate, no changes over time or between groups have been recorded, while the b* value increased from the sixth day of storage, reaching the highest value in the PE group (Figure 5B,C). Previous studies report variable data on shrimp color changes during storage as a function of time and the substances used for their preservation [8,38,52]. Senapati et al. [53] reported trends of L*, a* and b* coordinates similar to our results in white shrimp during chill storage.

### 3.3. Microbiological Analysis

**Figure 6:**
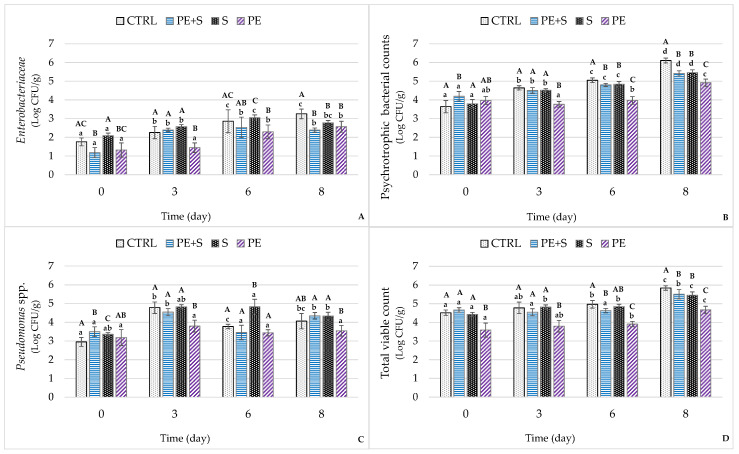
Microbiological changes during the storage (D0 = day of packaging, D3 = three days after packaging, D6 = six days after packaging and D8 = eight days after packaging) of shrimps (*n* = 5) treated with tap water (CTRL), 0.25% sodium metabisulfite tap water solution containing 1 g/L of phenols (PE + S), 0.5% sodium metabisulfite tap water solution (S), and tap water solution containing 2 g/L of phenols (PE). Different uppercase letters, within each day of storage, represent significant differences between groups (*p* < 0.05); different lowercase letters, within each group, represent significant differences between days of storage (*p* < 0.05). *Enterobacteriaceae* (**A**), psychrotrophic bacterial counts (**B**), *Pseudomonas* spp. (**C**) and total viable count (**D**).

The evolution of the microbiological profile of the shrimp samples during storage is shown in Figure 6. All the parameters analyzed increased over time (*p* < 0.001), with variable growth rates depending on the group and the evaluated criterion. In particular, the *Enterobacteriaceae* at day 0 presented a microbial load that ranged from 1.18 ± 0.13 Log CFU/g in the PE + S group to 1.76 ± 0.13 Log CFU/g in the S group. Subsequently, the counts progressively increased, exceeding 3 Log CFU/g in the CTRL samples after 8 storage days (Figure 6A). The final mean value recorded in this group was higher than in the others (*p* < 0.05 to *p* < 0.001), as was the growth rate (k) (44.6%/*d*, *p* < 0.001) (Table S3). From this point of view, the highest growth rate (k) was found for psychrotrophic bacteria (70.9%/*d*, *p* < 0.001) in the CTRL samples, determining higher microbial increases than in the other groups (*p* < 0.001; Figure 6B). In contrast, the most remarkable reduction in k for this microbic flora was recorded in the PE samples (27.2%/*d*, *p* < 0.001; Table S3). Therefore, at the end of conservation, the mean count in the control group was significantly higher, while it was the lowest in the PE group (6.10 ± 0.06 vs. 4.92 ± 0.06 Log CFU/g; *p* < 0.001). In terms of shelf-life, this is a rather important result, because bacteria belonging to this category play a primary role in the alteration processes of food stored under refrigeration conditions [40].

In the current work, the correlation analysis shows particularly high coefficients between psychrotrophic bacteria and both TBARS and TVB-N (*p* < 0.01) (Table S2), indices that are notoriously associated with the degradation of the lipid and protein components, respectively.

The antibacterial action of natural antioxidants is well known, while the synergistic effect of individual compounds and bacterial sensitivity is still relatively unrecognized. In the current work, in fact, PEOVW, either alone or combined with sodium metabisulfite, did not lead to significant reductions in *Pseudomonas* spp. compared to control (*p* < 0.05; Figure 6C). In fact, previous in vitro studies using a PEOVW similar to the one tested in this work found that strains belonging to this genus are not very sensitive to the bactericidal action of phenolic compounds [54]. Pereira et al. [55] reached the same conclusions when evaluating the in vitro activity against several microorganisms of phenolic compounds derived from olive leaves.

Our results are overall consistent with those recorded in a previous study by Miraglia et al. [18], which showed a reduction in TVC and *Enterobacteriaceae* count in fresh salmon treated with phenolic extract derived from olive vegetation water (Figure 6D). Similar results have also been obtained in shrimps treated with rosemary extract [52], cashew leaf [31], green tea [15] and grape seed extracts [16]. Menchetti et al. [56], in agreement with our evidence, highlighted the bactericidal action of PEOVW on *S.* Enteritidis experimentally inoculated in mayonnaise.

### 3.4. Melanosis

**Figure 7:**
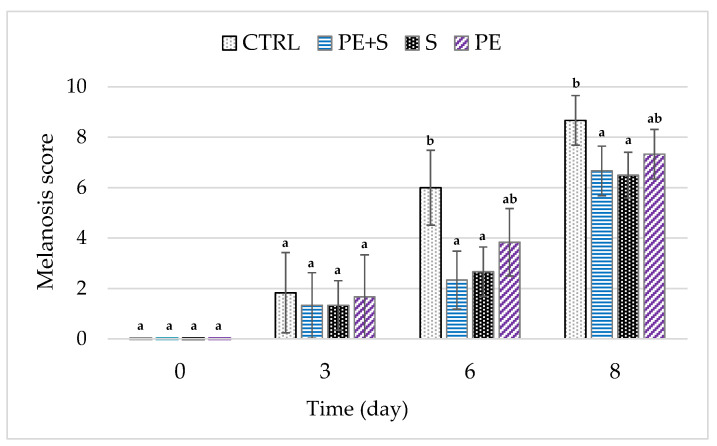
Melanosis score during storage of shrimps treated with tap water (CTRL), 0.25% sodium metabisulfite tap water solution containing 1 g/L of phenols (PE + S), 0.5% sodium metabisulfite tap water solution (S), and tap water solution containing 2 g/L of phenols (PE). Different letters, within each day of storage, represent significant differences between groups (*p* < 0.05).

Melanosis is caused by biochemical reactions catalyzed by endogenous polyphenol-oxidase enzymes that remain active even at refrigeration temperatures [31]. These reactions generate a build-up of high-molecular-weight black pigments (black spots) visible in the shell [57], with a consequent decrease in the value of the shrimps [57]. Changes in melanosis during storage time are reported in Figure 7.

No melanosis was found in all groups at day 0, but as the days went by there was a progressive increase, regardless of treatment. However, no differences were recorded between groups until the sixth day (*p* > 0.05), when the S and PE + S samples received the lowest scores and the CTRL group the highest one (*p* < 0.05). These differences remained unvaried until the end of the observations period, resulting in the following final scores: S = 6.50, PE + S = 6.67, PE = 7.33 and CTRL = 8.67. The results obtained agree with those reported by Yuan et al. [42], in which the melanosis scores of the Pacific white shrimp treated with pomegranate peel extract were lower than those untreated, but not lower than those treated with sodium metabisulfite.

Sulfites are reducing agents that prevent enzymatic browning through different processes, including the reduction of reactive ortho-quinone into colorless diphenol, and reacting irreversibly with polyphenol-oxidase (PPO) by completely inactivating it [4]. Moreover, the PE + S group obtained, at the end of the treatment, a score close to that obtained by the S group. This result shows that the effect of half a dose of sodium metabisulfite can be combined with the antioxidant activity of the phenolic compounds of the extract, thus significantly delaying the oxidation reactions.

## 4. Conclusions

Preserving the foodstuffs’ quality during storage, without adding synthetic additives, is a strategy that the industry in this sector, particularly the fish industry, has been trying to pursue in recent years. To date, a great part of the research has been oriented towards the use of plant-derived bioactive compounds, which combine antioxidant, antimicrobial and health properties. The olive vegetation water phenolic extract, as a natural additive tested in this study on pink shrimp, showed several interesting implications. In particular, it was able to delay the alterative phenomena by slowing down lipid oxidation, microbial development and the volatile nitrogen compounds’ formation, in a manner proportionally effective to the dose of extract used. 

The effects on melanosis formation were less evident. Phenolic compounds alone, at a concentration of 2 g/L, showed a marginal, yet not significant (*p* < 0.05), inhibitory effect on the black spots’ progression. However, the treatment with 1 g/L of phenols, added to a 0.25% sodium metabisulfite solution, was able to delay melanosis with an efficacy equal to sodium metabisulfite solution alone at a concentration of 0.5%, which is normally used by the manufacturers for anti-melanosis in shrimp. This result is very promising, given the well-known adverse reactions associated with the sulfites additives. Indeed, being able to treat foods with lower quantities of sulfites, combining them with the antioxidant power of natural compounds, would allow one to obtain, on the one hand, a healthier product and, on the other hand, a product acceptable to the consumer in the same way as one treated only with sulfites. In this regard, several experimental trials have been conducted aimed at reaching more stability in the PEOVW over time. In particular, systems such as (i) its storage at −20 °C, (ii) its microencapsulation in silica and lipid gels of the phenolic concentrate derived from olive vegetation water and its further storage at fridge temperatures, and (iii) a spray drying process after the phenolic concentrate’s immobilization in maltodextrins, and further storage of the powder at room temperature, have been successfully tested. Thus, this natural additive could be made available all year round.

## Supplementary Materials

The following are available online at https://www.mdpi.com/2304-8158/9/11/1647/s1, Figure S1: HPLC chromatogram of phenolic extract purified from the concentrate of vegetation water recorded with DAD at 278 nm. Peak numbers: 1, 3,4-DHPEA (Hydroxytyrosol); 2, *p*-HPEA (Tyrosol); 3, Verbascoside; 4, 3,4-DHPEA-EDA (Oleacin), Table S1: Quali-quantitative composition of the OVW (Servili et al., 2011), Table S2: Correlation analysis (Spearman rank correlation coefficient, *ρ*), Table S3. Parameter estimates of the log-linear models fitted for shrimps treated with tap water (CTRL), 0.25% sodium metabisulphite tap water solution containing 1 g/L of phenols (PE + S), 0.5% sodium metabisulphite tap water solution (S) and tap water solution containing 2 g/L of phenols (PE).
