# Supplementary material for: Effect of an Olive Vegetation Water Phenolic Extract on the Physico-Chemical, Microbiological and Sensory Traits of Shrimp (Parapenaeus longirostris) during the Shelf-Life
Source: Foods. 2020 Nov 11;9(11):1647. doi: 10.3390/foods9111647 (PMC7696022; doi:10.3390/foods9111647)
Supplement: Supplementary file 1 [file foods-09-01647-s001.pdf]

foods

MDPI

Supplementary Materials Olive Vegetation Water; Quali-Quantitative Determination and Recovery of Phenols in PEOVW

1. Olive Vegetation Water (OVW)

According to Servili et al. [1], OVW consists of an emulsion composed of oil, mucilage and pectin. Generally, OVW pH ranges from 4.5 to 6 and includes 3–16% organic compounds, of which 1–8% are sugars, 1.2–2.4% are nitrogen-containing compounds and 0.34–1.13% are phenols (around 5 g/L; Table S1). According to Niaounakis and Halvadakis [2], its biochemical oxygen demand (BOD5) value generally ranges between 35 and 110 g/L, and its chemical oxygen demand (COD) ranges from 40 to 195 g/L.

Table S1. Quali-quantitative composition of the OVW (Servili et al., 2011) [1].

Compound	Concentration (g/L)
3,4-DHPEA	0.01 ± 0.001
p-HPEA	0.02 ± 0.004
3,4-DHPEA-EDA	4.1 ± 0.1
Verbascoside	0.7 ± 0.1
Total phenols	4.9 ± 0.2

Mean values ± standard deviation

2. Quali-quantitative Determination of Phenols in PEOVW

According to Esposto et al. [3], to extract phenols from PE, 50 mg of the sample were dissolved in 5 mL of methanol, filtered with a 0.2-lm PVDF syringe filter (Whatman, Clifton, NJ) and injected into the high-performance liquid chromatograph (HPLC). The analysis of polyphenols contained in the PEOVW was conducted with direct injection dissolving 1 g of oil in 5 mL of acetone, and then the solution was filtered through a polyvinylidene fluoride (PVDF) syringe filter (0.2 μm). HPLC analysis was performed using an Agilent Technologies system model 1100 consisting of a vacuum degasser, a quaternary pump, an autosampler, a thermostatted column compartment, a diode array detector (DAD), and a fluorescence detector (FLD) controlled by ChemStation (Agilent Technologies, Palo Alto, CA. USA) and used for the elaboration of chromatographic data. A Spherisorb ODS-1 column was used to evaluate the phenolic compounds; the mobile phase consisted of 0.2% acetic acid (pH 3.1) in water (solvent A)/methanol (solvent B) at a flow rate of 1 mL/min, and the gradient changed as follows: 95% A for 2 min, 75% A in 8 min, 60% A in 10 min, 50% A in 16 min, and 0% A in 14 min and maintained for 10 min. Following the re-equilibration of the initial conditions, equilibration was reached in 13 min; the total running time was 73 min. All phenolic compounds, except lignans, which were detected by FLD, operated at an excitation wavelength of 280 nm and emission at 339 nm, and were detected by DAD at 278 nm.

3. PEOVW Recovery

According to Servili et al. [4], a crude phenolic concentrate (CPC) from olive vegetation water (OVW) was previously obtained by fresh OVW (worked within 24 h from the virgin olive oil extraction process).

The extraction of CPC was carried out at 20°C under N_2 atmosphere, via a three step membrane filtration processes including microfiltration, ultrafiltration and reverse osmosis. Microfiltration (cut-

off 0.1–0.3 μm) was through a polypropylene tubular membrane (total area of 8 m^2). Ultrafiltration (cut-off 7 kDa) was through two spiral membranes of polyamide and traces of polysulfone (total area of 16 m^2). A spiral (thin-film, TFM), consisting of DurasanTM and polysulfone (total area of 9 m^2), was used for reverse osmosis. The membrane had the capacity to retain molecules with a molecular weight of ca. 100 Da. All membranes were purchased from Permeare s.r.l. (Milan, Italy). The olive vegetation water phenolic extract (PEOVW) was recovered from CPC by liquid/liquid extraction (LLE). The conditions for LLE were as follows: 100 mL of CPC (19.7 g/L of total phenols) was homogenized with ethyl acetate (50 mL) for 1 min, the organic phase was recovered and the aqueous residue was subjected to a second extraction. After saturation with sodium sulphate to remove water, the collected organic phase was filtered through a paper filter and the solvent was evaporated. The extract was washed three times with ethanol and evaporated to remove residual ethyl acetate. The purified phenolic extract (Figure S1) obtained was dissolved in 5 mL of ethanol, which was then evaporated using a flow of nitrogen.

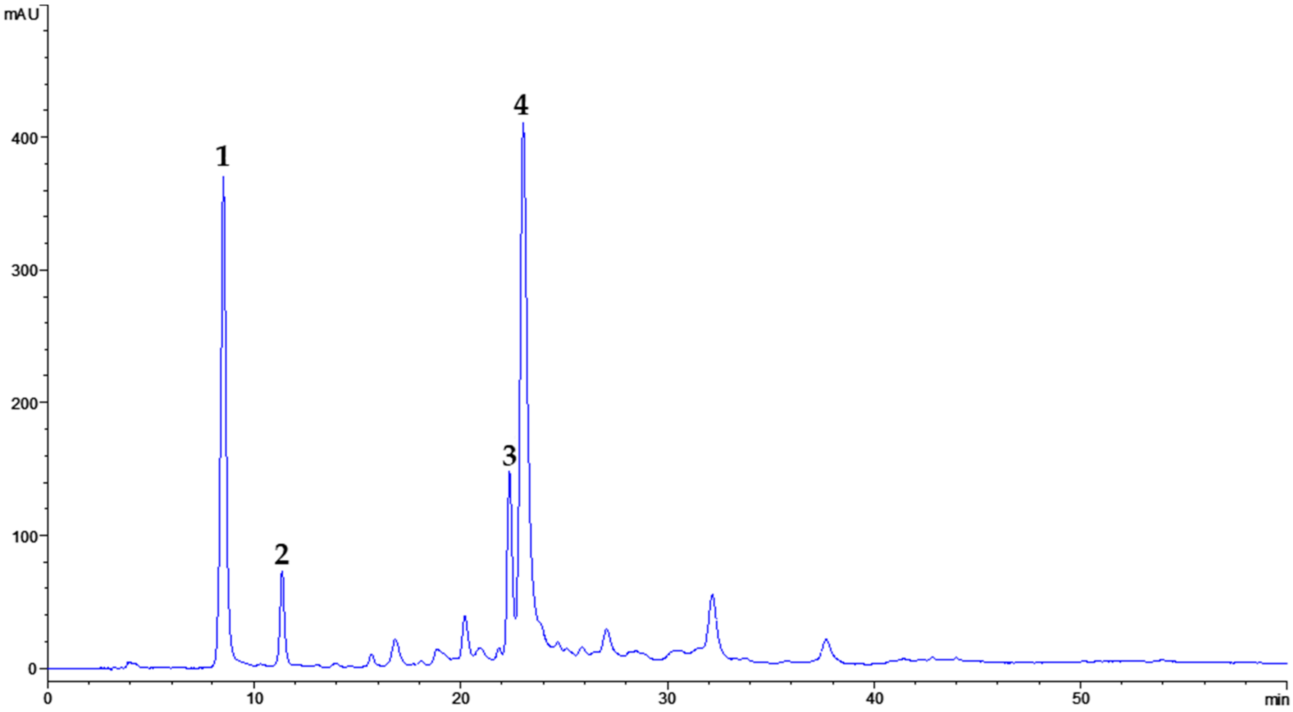

Figure S1. HPLC chromatogram of phenolic extract purified from the concentrate of vegetation water recorded with DAD at 278 nm. Peak numbers: 1, 3,4-DHPEA (Hydroxytyrosol); 2, *p*-HPEA (Tyrosol); 3, Verbascoside; 4, 3,4-DHPEA-EDA (Oleacin).

Table S2. Correlation analysis (Spearman rank correlation coefficient, ρ).

	Polyphenols	pH	Melanosis	TVB-N	TBARS	L*	a*	b*	*Enterobacteriaceae* (Log CFU/g)	Total viable count (Log CFU/g)	Psychrotrophic bacterial counts (Log CFU/g)
**pH**	-.253										
**Melanosis**	-.152	.204									
**TVB-N**	-.214	**.752****	.420								
**TBARS**	**-.634***	.432	.435	**.643***							
**L***	-.377	.357	.107	.303	.364						
**a***	.384	-.012	.208	-.052	-.503	.396					
**b***	-.082	-.131	.345	-.179	-.147	.174	.453				
***Enterobacteriaceae* (Log CFU/g)**	**-.692****	.339	.328	.422	**.722****	.286	-.416	.243			
**Total viable count (Log CFU/g)**	-.338	**.569***	.028	**.856****	**.692***	.274	-.268	-.406	**.477***		
**Psychrotrophic bacterial counts (Log CFU/g)**	**-.538****	**.556***	.279	**.850****	**.720****	.400	-.222	-.159	**.586****	**.871****	
***Pseudomonas* spp. (Log CFU/g)**	-.045	.380	**-.457***	.410	.077	**.580***	.169	-.266	.018	**.559***	.391

** Correlation is significant at the 0.01 level (2-tailed); * Correlation is significant at the 0.05 level (2-tailed). Correlation coefficients higher than │0.500│ are in bold.

Table S3. Parameter estimates of the log-linear models fitted for shrimps treated with tap water (CTRL), 0.25% sodium metabisulphite tap water solution containing 1 g/L of phenols (PE + S), 0.5% sodium metabisulphite tap water solution (S) and tap water solution containing 2 g/L of phenols (PE).

Bacteria	Group	K (%/d)	SE	P value	R^2	τ (d)
Enterobacteriaceae	CTRL	44.6	6.8	<0.001	0.703	2
	PE + S	37.3	9.4	0.001	0.483	2
	S	21.9	4.8	<0.001	0.539	3
	PE	39.3	8.6	0.003	0.597	2
Total viable count	CTRL	36.6	4.5	<0.001	0.770	2
	PE + S	23.1	4.9	<0.001	0.515	3
	S	28.0	3.1	<0.001	0.793	2
	PE	30.9	6.6	<0.001	0.551	2
Psychrotrophic bacterial counts	CTRL	70.9	4.7	<0.001	0.916	1
	PE + S	35.1	4.1	<0.001	0.772	2
	S	47.5	3.3	<0.001	0.901	1
	PE	27.2	6.7	0.001	0.439	3
Pseudomonas spp.	CTRL	26.1	11.7	0.042	0.263	3
	PE + S	20.4	8.5	0.028	0.242	3
	S	30.1	9.0	0.003	0.370	3
	PE	8.1	9.1	0.388	0.054	9

K= growth rate constant; SE= standard error for k; R^2 = coefficient of determination; τ (d) = generation time.

References

1. Servili, M.; Esposto, S.; Veneziani, G.; Urbani, S.; Taticchi, A.; Di Maio, I.; Selvaggini, R.; Sordini, B.; Montedoro, G.F. Improvement of bioactive phenol content in virgin olive oil with an olive-vegetation water concentrate produced by membrane treatment. *Food Chem.* **2011**, doi:10.1016/j.foodchem.2010.07.042.
2. Niaounakis, M.; Halvadakis, C.P. Olive Processing Waste Management. *Lit. Rev. Pat. Surv. 2nd Ed.* **2006**.
3. Esposto, S.; Taticchi, A.; Di Maio, I.; Urbani, S.; Veneziani, G.; Selvaggini, R.; Sordini, B.; Servili, M. Effect of an olive phenolic extract on the quality of vegetable oils during frying. *Food Chem.* **2015**, *176*, 184-192, doi:10.1016/j.foodchem.2014.12.036.
4. Servili, M.; Rizzello, C.G.; Taticchi, A.; Esposto, S.; Urbani, S.; Mazzacane, F.; Di Maio, I.; Selvaggini, R.; Gobbetti, M.; Di Cagno, R. Functional milk beverage fortified with phenolic compounds extracted from olive vegetation water, and fermented with functional lactic acid bacteria. *Int. J. Food Microbiol.* **2011**, doi:10.1016/j.ijfoodmicro.2011.03.006.

© 2020 by the authors. Licensee MDPI, Basel, Switzerland. This article is an open access article distributed under the terms and conditions of the Creative Commons Attribution (CC BY) license (http://creativecommons.org/licenses/by/4.0/).
